# Factor structure of the Chinese version of Emotion Regulation Goals Scale

**DOI:** 10.3389/fpsyg.2024.1392879

**Published:** 2024-07-08

**Authors:** Shengdong Chen, Cheng Chi, Li Luo, Weiwei Zhu, Yi Chen, Tao Wang, Jiajin Yuan

**Affiliations:** ^1^School of Psychology, Qufu Normal University, Qufu, China; ^2^Department of Education Science, Neijiang Normal University, Neijiang, Sichuan, China; ^3^Institute of Brain and Psychological Science, Sichuan Normal University, Chengdu, Sichuan, China; ^4^Sichuan Key Laboratory of Psychology and Behavior of Discipline Inspection and Supervision, Sichuan Normal University, Chengdu, China

**Keywords:** emotion regulation goals, exploratory factor analysis, confirmatory factor analysis, reliability, validity

## Abstract

Recent studies in Western cultures suggested emotion regulation goals have important implications for mental health. This study aimed to test the factor structure of Emotion Regulation Goals Scale (ERGS) in a Chinese cultural context. Exploratory factor analysis (EFA) and confirmatory factor analysis (CFA) were first used to examine the factor structure of the ERGS, and then reliability and validity tests were conducted to examine the psychometric properties of the ERGS. Results showed that the original five-factor model demonstrated fit during both EFA and CFA, and was thus adopted for further psychometric analyses. Most of the five factors were significantly associated with emotion regulation tendencies and negative emotional outcomes (e.g., depression), except for the non-significant associations between pro-hedonic goals and expressive suppression, and pro-social and impression management goals with depression. The ERGS also showed good internal consistency and split-half reliability. However, the test-retest reliabilities varied substantially across the five factors. The pro-hedonic goal had a higher test-retest reliability, whereas the contra-hedonic, performance, pro-social, and impression management goals showed lower values, especially the latter two. In brief, the ERGS showed a promising five-factor structure in assessing emotion regulation goals in Chinese cultural context.

## Introduction

Emotion regulation goals refer to the reasons individuals engage in emotional regulation (Tamir, 2016), influencing the choice of regulation strategies (Millgram et al., 2019) and the regulation effects (Tamir et al., 2019), and closely linking to mental health (in terms of less depression, anxiety, and stress; Brandão et al., 2023). However, in the early research stages, researchers did not have a unified standard tool to measure individual emotion regulation goals. Various measures were used to evaluate emotion regulation goals, including single items from self-regulation questionnaires (Benita et al., 2019), self-generated items (Kalokerinos et al., 2017), and picture selection paradigms (Millgram et al., 2015; Li et al., 2019). These measures either have a broad focus or a specific focus on emotion regulation goals, leaving a challenge for determining structures of emotion regulation goals.

In order to standardize the measurement of individual emotion regulation goals, Eldesouky and English developed the Emotion Regulation Goals Scale (ERGS; Eldesouky and English, 2019a). The original version of the ERGS comprises five dimensions and 18 items. The five dimensions correspond to five typical emotion regulation goals: (1) pro-hedonic goal, indicating individuals seek to increase or maintain positive feelings by approaching positive stimuli or avoiding negative stimuli; (2) contra-hedonic goal, indicating individuals seek to increase or maintain negative feelings by approaching negative stimuli or avoiding positive stimuli; (3) performance goal, indicating individuals focus on work or study-related pursuits; (4) pro-social goal, indicating individuals seek to maintain or enhance the quality of interpersonal relationships; (5) impression management goals, indicating individuals seek to maintain or enhance the impression they create in the minds of others.

The Emotion Regulation Goals Scale has been translated into Portuguese (Brandão et al., 2022) and German (Wilms et al., 2021), demonstrating good reliability and validity. However, current studies validating the reliability and validity of the Emotion Regulation Goals Scale have primarily been conducted in Western cultural contexts. Previous research has indicated that cultural context has a significant impact on individuals’ tendencies and effectiveness in emotion regulation. For instance, expressive expression was associated with negative emotion regulation outcomes and greater cognitive and social costs (Butler et al., 2003; Gross and John, 2003), whereas was associated with adaptive regulatory outcomes in Eastern cultures (Chen et al., 2017). Therefore, in order to understand the potential similarities and differences in individuals’ emotion regulation goals across cultural backgrounds, investigating the applicability of ERGS in an Eastern cultural context is necessary.

To address this issue, we examined the factor structure and psychometric properties of the ERGS in a Chinese cultural context. Specifically, we tested the five-factor structure of the ERGS using exploratory factor analysis (EFA) and confirmatory factor analysis (CFA), tested the ERGS construct validity with relevant related theoretical constructs, i.e., emotion regulation tendencies and negative emotional outcomes (e.g., anxiety and depression). We finally tested the ERGS reliability in two samples.

In terms of the links between the emotion regulation goals and the other theoretically-related constructs, previous findings from western-culture were not fully consistent. Among the five emotion-regulatory goals, the pro-hedonic goal was reported most consistently to exist positive and moderate associations with cognitive reappraisal tendency and negative and moderate associations with negative emotional outcomes (e.g., anxiety and depression) across American and German samples (English et al., 2017; Eldesouky and English, 2019b; Wilms et al., 2020). The positive associations of contra-hedonic goal, prosocial goal and impression management goal with expressive suppression tendency were weak and unstable (Eldesouky and English, 2019b; Wilms et al., 2020; Brandão et al., 2022). Considering the cross-cultural consistency in the moderate associations found between pro-hedonic goals and emotional constructs, we expect the associations between the pro- and contra-hedonic goals and emotional constructs (e.g., cognitive reappraisal) would also exist in a Chinese culture context.

## Materials and methods

### Participants

Participants recruited through the Internet Aids at one Chinese university. Four samples and a total of 2,532 participants were recruited during the questionnaire revision process. Sample A consisted of 1,181 undergraduates and was collected online. Sample A was used for item analysis, EFA, structural validity analysis, criterion-related validity analysis, internal consistency, and split-half reliability analysis. Sample B consisted of 651 undergraduates (M_age_ = 18.20, SD = 0.67; male/female = 144/507) and was used for CFA. Sample C consisted of 130 undergraduates (M_age_ = 19.14, SD = 1.25; male/female = 42/88) and was used for test-retest reliability analysis. The time interval between the two measurements was 1 week. The sample C data were collected offline. Sample D consisted of 500 undergraduates independent of the above samples (M_age_ = 19.37, SD = 1.20; male/female = 75/8,425) for validating the CFA results.

Data was cleaned after collection. For example, participants with excessively short response times and those who inaccurately answered deception items (e.g., “I never sleep”) were excluded. Finally, 917 valid datasets were retained (M_age_ = 19.48, SD = 1.26; male/female = 125/792) for Sample A. No participants were excluded for Sample B. Two data points were missing during the second measurement, resulting in a final valid dataset of 128 individuals for Sample C. This study was not preregistered. Data of this study would be available upon reasonable request to the corresponding author.

### Measures

#### Chinese version of Emotion Regulation Goals Scale

This study utilized the Emotion Regulation Goals Scale (ERGS) developed by Eldesouky and English (2019a). With authorization from the original authors, the scale was translated, maintaining the original 7-point rating system where 1 represents “never” and 7 represents “always.” The 18 original items corresponded to five dimensions as follows: (1) pro-hedonic goal (items 1–3); (2) contra-hedonic goal (items 4–6); (3) performance goal (items 7–9); (4) pro-social goal (items 10–14); (5) impression management goals (items 15–18).

The questionnaire underwent translation using the revised Brislin translation method (Jones et al., 2001). Two psychology graduate students independently translated the original English version into Chinese to create an initial draft. Two additional psychology graduate students were invited to back-translate the questionnaire into English. Subsequently, a panel of four experts conducted a conceptual review of the two back-translated versions in a face-to-face interview, ensuring consistency with the original version. The expert panel then merged and revised the translations to obtain a revised version. This process was repeated until the entire team reached a consensus without any disputes regarding the back-translated versions and the original items. Finally, 30 undergraduate students independently pilot-tested the revised questionnaire to ensure semantic clarity. Based on the feedback received, adjustments were made to items with ambiguity or comprehension difficulties, resulting in the final Chinese version of the Emotion Regulation Goals Scale.

#### Emotion regulation questionnaire (ERQ)

The ERQ, developed by Gross and John (2003), is a classic questionnaire designed to measure individuals’ daily emotion regulation habits. Higher scores on the ERQ often reflect a higher tendency for cognitive reappraisal (Gross and John, 2003) and expressive suppression (Chen et al., 2017). The ERQ comprises two subscales, one measuring individuals’ tendency to use cognitive reappraisal strategies and the other measuring the tendency to use expressive suppression strategies. Participants rate ERQ items on a 7-point scale (1 = strongly disagree, 7 = strongly agree). Cronbach’s alpha coefficients for cognitive reappraisal and expressive suppression, calculated based on Sample A, were 0.85 and 0.57, respectively.

#### Depression anxiety stress scales (DASS-21)

The DASS-21is designed to assess individuals’ self-reported negative emotional states in the domains of depression, anxiety, and stress (Henry and Crawford, 2005; Gong et al., 2010). Previous research indicates the applicability of this scale for auxiliary diagnosis and outcome monitoring in clinical settings, as well as its suitability as a mental health screening tool in non-clinical environments (Henry and Crawford, 2005). Participants are required to rate the extent to which they identify with the symptom descriptions for each item on a 5-point scale (0 = does not apply, 4 = applies always). The Cronbach’s alpha coefficients for the depression, anxiety, and stress subscales, calculated based on Sample A, were 0.86, 0.74, and 0.85, respectively.

### Statistical methods and tools

Correlation analysis, EFA, and reliability analysis were conducted using SPSS 23.0. Confirmatory factor analysis was performed using Amos 20.0. The EFA parallel analysis (Horn, 1965) was conducted using Jamovi Software (Şahin and Aybek, 2019).

## Results

### Item analysis

We employed the critical ratio method to conduct item analysis on the data from Sample A. Specifically, this study first calculated the critical ratio (CR) for each item on the Emotion Regulation Goals Scale. Subsequently, the CR total score for all items was computed. Next, the total scores were arranged in descending order, with the top 27% considered as the high-score group and the bottom 27% as the low-score group. Finally, independent sample *t*-tests were conducted to examine whether the differences in CR values for each item between the high-score and low-score groups reached significance. The results showed that, except for the three items in the contra-hedonic dimension, the CR values for other items showed significant differences between high and low groups, with *t*s > 3, *p*s < 0.05. For the three items in the contra-hedonic goal dimension, CR values did not differ significantly between high and low groups, with 0.22 > *t*s > −1.1, *p*s > 0.50. These results suggest that, except for items related to contra-hedonic goals, all other items demonstrated good discriminant validity. However, items related to contra-hedonic goals were retained in subsequent analyses to maintain consistency with the structure of original ERGS.

### Validity analysis

#### Exploratory factor analysis

The structure of the 18-items ERGS was first explored using EFA in Sample A with Promax oblique rotation, because the emotion regulation goals were reported to be correlated (Eldesouky and English, 2019a). Bartlett’s test results showed the values of Kaiser-Meyer-Olkin (KMO) was significant and acceptable (KMO = 0.88, χ^2^ = 8,510.18, *p* < 0.001), indicating that the overall correlation structure is suitable for EFA. Subsequent factor analyses were conducted in a stepwise fashion to eliminate items until a stable factor solution emerged.

In terms of the criteria of item elimination, items with loadings < 0.7 and items with loadings > 0.7 on more than one factor were excluded because the factor loadings score of 0.7 (or above) is generally considered a good cut-off (Stevens, 2002; Field, 2013). Following this criterion, the original first item (“To experience fewer negative emotions, such as sadness”) with a factor loading below 0.7 was eliminated. The factor loading matrixes before and after item removal were provided in Supplementary Tables 1, 2.

**FIGURE 1:**
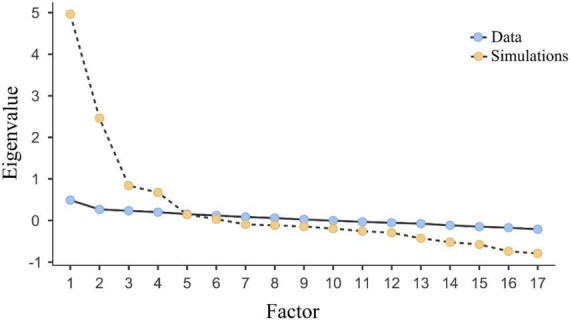
Scree plot of the EFA and parallel analysis.

Factor analysis was conducted for the second time on the remaining 17 items. According to the eigenvalue criterion, there were four factors with eigenvalues exceeding 1 (Kaiser, 1960), and one factor with eigenvalues exceeding 0.7 (Jolliffe, 1986). The extracted four and five factors cumulatively contributed to 62.36 and 66.42% of the total variance, respectively. On the other hand, the point of inflection of the scree plot occurred at factor five (Figure 1). Taken together, five factors were retained in accordance with the criteria of conceptual coherence, eigenvalues above 0.7, and the cut-off point of the scree plot (Supplementary Table 3).

#### Confirmatory factor analysis

**FIGURE 2:**
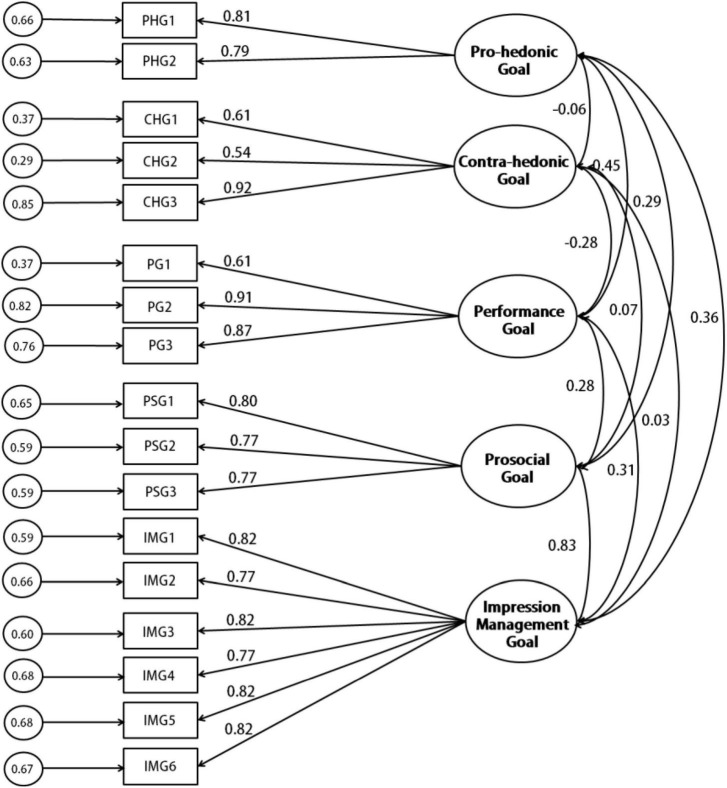
CFA of the final 17-items Emotion Regulation Goals Scale.

To confirm the structure of the ERGS, we tested the five-factor CFAs using maximum likelihood estimation in AMOS in Sample B and D, respectively. Previous research suggests that the Tucker-Lewis index (TFI) and comparative fit index (CFI) values of 0.9, a Goodness-of-Fit Index (GFI) value of 0.9, a root mean square error of approximation (RMSEA) value of 0.6 or less were considered to indicate acceptable model fit (Schumacker and Lomax, 2016). According to these standards of CFA model fitting index, the results of both CFA tests showed that the fitting indices of the five-factor model reached an acceptable, but not good standard (Table 1), suggesting the stability of the five-factor structure of the ERGS (Figure 2).

**TABLE 1:** Fit Indices for confirmatory factor analysis models.

Sample	Models	χ^2^(df)	χ^2^/df	GFI	CFI	AGFI	TLI	AIC	RESMA
Sample B	Five-factor	635.05 (109)	5.83	0.92	0.94	0.88	0.92	723.05	0.073
Sample D	Five-factor	269.98 (109)	3.39	0.91	0.94	0.88	0.93	457.98	0.069

#### Structural validity analysis

To explore the structural validity of the ERGS, Pearson correlation analyses were conducted between the five factors in Sample A. Results showed that most correlations were statistically significant except for certain correlations of contra-hedonic goals with pro-hedonic and impression management goals (Table 2).

**TABLE 2:** Structural validity of the emotion regulation scale.

	Pro- hedonic	Contra-hedonic	Performance	Prosocial	Impression management
Pro- hedonic	1				
Contra-hedonic	−0.004	1			
Performance	0.34**	−0.18**	1		
Prosocial	0.23**	0.07	0.24**	1	
Impression management	0.31**	0.05	0.26**	0.72**	1

** means *p* < 0.001.

#### Criterion validity analysis

Because there are correlations among the five emotion regulation goals, regression methods were used to establish criterion validity for controlling covariates. Multiple regressions were conducted with the goals as simultaneous predictors of each emotion regulation strategy and each negative emotional outcome based on Sample A data.

Results showed that individuals endorsing more pro-hedonic, performance and prosocial goals, and less contra-hedonic goals reported higher tendencies of cognitive reappraisal (Table 3). In contrast, those endorsing more contra-hedonic and performance goals reported higher tendencies of expression suppression. Furthermore, individuals who endorsed more contra-hedonic and impression management goals, and less pro-hedonic goals experienced higher-levels of negative emotions including stress, anxiety, and depression. Those endorsing less performance goal reported higher-levels of anxiety and depression.

**TABLE 3:** Regression analysis of emotion regulation goals predicting emotion regulation strategy use and negative emotion outcome.

	Pro-hedonic	Contra-hedonic	Performance	Prosocial	Impression management
**Emotion regulation strategies**					
Cognitive reappraisal	0.17**	−0.11**	0.32**	0.12**	0.04
Expression suppression	−0.05	0.17**	0.08*	0.07	0.08
**Negative emotion outcomes**					
Stress	−0.16**	0.20**	−0.05	−0.05	0.19**
Anxiety	−0.15**	0.21**	−0.07*	−0.01	0.16**
Depression	−0.23**	0.25**	−0.07*	−0.04	0.15**

Values reflect standardized beta coefficients. * and ** mean *p* < 0.05 and *p* < 0.001, respectively.

#### Reliability analysis

#### Internal reliability

Internal reliability was measured by Cronbach’s alpha coefficients based on the data from Sample A. Results showed that the Cronbach’s alpha coefficients for pro-hedonic goals, contra-hedonic goals, performance goals, pro-social goals, and impression management goals were 0.78, 0.71, 0.82, 0.82, and 0.92, respectively.

#### Test-retest reliability

Test-retest reliability measured by the intraclass correlation coefficient (ICC; Aldridge et al., 2017) was analyzed based on the data from Sample C. Results showed that the ICCs for pro-hedonic goals, contra-hedonic goals, performance goals, pro-social goals, and impression management goals were 0.80, 0.59, 0.59, 0.50, and 0.42, respectively.

## Discussion

A growing body of studies confirms that the formulation of emotion regulation goal determines the choice of emotion regulation strategies (Millgram et al., 2019) and is closely related to the severity of individual emotional disorder symptoms (Millgram et al., 2015; Li et al., 2019). However, the currently developed measurement scale of emotion regulation goal (i.e., ERGS) have only been validated in Western cultural contexts, and their applicability in the Chinese cultural context remains unknown. This study, for the first time, examined the structure, reliability and validity of the ERGS in a Chinese cultural context.

First, item analysis revealed that except the relatively weak discriminant validity of contra-hedonic dimension items, most ERGS items had good discriminant validity. Consistently, the structural validity analysis also indicated that the contra-hedonic goal had the smallest correlations with other goals. These findings suggest that the contra-hedonic goals might be independent of the other emotion-regulatory goal on certain dimension, i.e., goal orientation (Freund et al., 2012). Specifically, only the contra-hedonic goals orient toward negative expected outcomes, whereas all the other goals orient toward positive expected outcomes. However, the current ERGS were developed based on the instrumental account that mainly stress on the goal benefits rather than the goal valence (Tamir, 2009). The theoretical framework of emotion-regulatory goals may need to be modified to better explain the structure of emotion regulatory goals.

In terms of the structure of current ERGS, 17 EFA items were extracted as four factors. Among them, there were three factors (pro-hedonic, contra-hedonic, and performance goals) consistent with the original scale, demonstrating cross-cultural consistency. From an evolutionary and cultural perspective, these three emotion regulation goals may be a self-regulatory mechanism that humans have developed to adapt to their environment, primarily influenced by human genes (Smederevac et al., 2023) and therefore less influenced by external cultural backgrounds. Moreover, though pro-social goals and impression management goals were extracted as a single factor in the first-time EFA, the second-time EFA extracted their items as two highly-correlated factors. Compared to previous research (*r* = 0.52) (Eldesouky and English, 2019a), there was a stronger correlation (*r* = 0.72) within these two social goals (Martini, 2011) or extrinsic goals (Grouzet et al., 2005).

Based on the existing two-dimensional goal model, compared to self-oriented emotion regulation goals aimed at satisfying one’s own needs (e.g., hedonic goals), other-oriented emotion regulation goals aim at meeting the needs of others rely more on social perception and social expectations, and are more likely to be influenced by external cultural backgrounds. Compared to Western individualistic culture, Chinese culture is a typical collectivist culture. A higher collectivist tendency is not only correlated with higher levels of prosocial behavior (Zhang and Han, 2023), but also leads individuals in this cultural context to use impression management strategies that focus more on interdependent relationships rather than self-characteristics (Chen, 2010). In light of the current research findings, one likely explanation is that Chinese culture has not altered the ERGS factor structure, but strengthened the connection strength between social goals aimed at others.

Consistent with previous research, structural validity analysis revealed the weakest correlation between hedonic and contra-hedonic goals, and the strongest correlation between the two social goals (Eldesouky and English, 2019a). Consistent with our hypothesis, criterion-related validity analysis showed emotion regulation goals predicting emotion regulation tendencies and negative emotional outcomes, especially for hedonic goals. Consistent with our findings, impaired motivational functions have been also reported to be closely correlated with maladaptive regulatory strategies (Millgram et al., 2015) and depressive symptoms in Western cultures (Becerra et al., 2013; Robson et al., 2023). These findings suggest that the Chinese ERGS have a promising constructive validity.

Reliability analysis showed that internal consistency for all five goals reaching acceptable levels. However, the test-retest reliability of five goals varied to a lager extent. Only test-retest reliability of pro-hedonic goal reached a good level, whereas the other goals only reached poor or moderate levels. One possible explanation for this phenomenon is that the five emotion regulation goals may be differentiated based on characteristics and states. The pro-hedonic goal tends to be a more inherent quality and therefore demonstrates greater consistency over time. On the other hand, other emotion regulation goals may be more susceptible to environmental influences, resulting in lower levels of stability over an extended period (Wilms et al., 2020).

Two important limitations should be acknowledged. First, all samples were university student samples. Future research should include non-college samples to test the generalizability of ERGS structure. Second, though the five-factor structure of the ERGS is replicated in a Chinese context, the origin of the ERGS was still ultimately developed in the Western culture. Future research could benefit from a bottom-up approach by actually creating the initial scale in a non-Western context first and then determining whether the same goal factors would arise.

In conclusion, our findings suggest that the overall five-factor structure of the ERGS remains stable in a Chinese culture, and its reliability and validity reached acceptable standards. However, our findings also suggest that there are certain distinctions among the five emotion regulation goals in their psychometric properties, which should be taken into consideration when applying ERGS as a tool for measuring emotion regulation goals in Chinese context.

## Data availability statement

The raw data supporting the conclusions of this article will be made available by the authors, without undue reservation.

## Ethics statement

The studies involving humans were approved by the Qufu Normal University Ethics Committee. The studies were conducted in accordance with the local legislation and institutional requirements. The participants provided their written informed consent to participate in this study.

## Author contributions

SC: Writing – original draft, Writing – review & editing. CC: Data curation, Formal analysis, Writing – review & editing. LL: Formal analysis, Writing – review & editing. WZ: Data curation, Resources, Writing – review & editing. YC: Data curation, Formal analysis, Writing – review & editing. TW: Conceptualization, Funding acquisition, Supervision, Writing – review & editing. JY: Supervision, Writing – review & editing.
