# Supplementary material for: Factor structure of the Chinese version of Emotion Regulation Goals Scale
Source: Front Psychol. 2024 Jul 8;15:1392879. doi: 10.3389/fpsyg.2024.1392879 (PMC11292033; doi:10.3389/fpsyg.2024.1392879)
Supplement: Supplementary file 1 [file Table_1.DOCX]

***Supplementary Material***

**S-Table 1 Four-Factor Loading Matrix before item 1 removed (N_A_ =917)**

| *ERGS Item* | Factor | | | |
| --- | --- | --- | --- | --- |
|  | 1 | 2 | 3 | 4 |
| 14.为了与他人保持亲密关系？(PSG) | **0.83** | 0.06 | 0.06 | 0.12 |
| 16.为了避免给他人留下一个令人不快的印象？(IMG) | **0.81** | 0.15 | -0.05 | 0.08 |
| 13.为了使他人开心？(PSG) | **0.81** | -0.05 | 0.10 | 0.16 |
| 17.为了得到他人的认可？(IMG) | **0.80** | 0.10 | -0.03 | 0.17 |
| 18.为了给他人留下一个好的印象？(IMG) | **0.79** | 0.19 | -0.11 | 0.09 |
| 15.为了避免被他人拒绝？(IMG) | **0.78** | 0.06 | 0.06 | 0.12 |
| 10.为了使他人感到舒适？(PSG) | **0.75** | 0.12 | 0.04 | 0.01 |
| 12.为了避免与他人疏远？(PSG) | **0.75** | 0.14 | 0.62 | 0.05 |
| 11.为了避免破坏他人的心情？(PSG) | **0.71** | 0.10 | 0.05 | -0.06 |
| 8.为了能够专注于自己的工作或正在进行的事情？(PG) | 0.13 | **0.86** | -0.13 | 0.17 |
| 7.为了避免被自己的情绪感受所干扰？(PG) | 0.10 | **0.81** | -0.03 | -0.08 |
| 9.为了能够保持对所进行工作的专注？(PG) | 0.18 | **0.80** | -0.15 | 0.22 |
| 1.为了感受更少的消极情绪（如生气，悲伤）？(PHG) | 0.08 | 0.48 | 0.02 | 0.13 |
| 6.为了持续感受消极情绪（如生气，悲伤）？(CHG) | 0.05 | -0.17 | **0.85** | -0.04 |
| 4.为了感受更多的消极情绪（如生气，悲伤）？(CHG) | 0.09 | -0.08 | **0.77** | -0.01 |
| 5.为了感受更少的积极情绪（如高兴，满足）？(CHG) | -0.03 | 0.04 | **0.76** | 0.05 |
| 3.为了持续感受积极情绪（如高兴，满足）？(PHG) | 0.20 | 0.15 | 0.05 | **0.86** |
| 2.为了感受更多的积极情绪（如高兴，满足）？(PHG) | 0.13 | 0.25 | -0.03 | **0.85** |

NOTE: PHG = pro-hedonic goals; CHG = contra-hedonic goals; PG = performance goals; PSG = pro-social goals; IMG = impression management goals. Boldface indicates items’ scale membership as determined by factor loading.

**S-Table 2** **Four-Factor Loading Matrix after item 1 removal (NA =917)**

| *ERGS Item* | Factor | | | |
| --- | --- | --- | --- | --- |
|  | 1 | 2 | 3 | 4 |
| 14.为了与他人保持亲密关系？(PSG) | **0.83** | 0.05 | 0.06 | 0.12 |
| 16.为了避免给他人留下一个令人不快的印象？(IMG) | **0.81** | 0.13 | -0.05 | 0.09 |
| 13.为了使他人开心？(PSG) | **0.81** | -0.06 | 0.10 | 0.16 |
| 17.为了得到他人的认可？(IMG) | **0.80** | 0.08 | -0.03 | 0.18 |
| 18.为了给他人留下一个好的印象？(IMG) | **0.80** | 0.17 | -0.11 | 0.10 |
| 15.为了避免被他人拒绝？(IMG) | **0.78** | 0.04 | 0.06 | 0.12 |
| 10.为了使他人感到舒适？(PSG) | **0.75** | 0.13 | 0.06 | 0.06 |
| 12.为了避免与他人疏远？(PSG) | **0.75** | 0.12 | 0.05 | 0.02 |
| 11.为了避免破坏他人的心情？(PSG) | **0.71** | 0.11 | 0.06 | -0.06 |
| 8.为了能够专注于自己的工作或正在进行的事情？(PG) | 0.13 | **0.87** | -0.12 | 0.21 |
| 9.为了能够保持对所进行工作的专注？(PG) | 0.18 | **0.82** | -0.13 | 0.26 |
| 7.为了避免被自己的情绪感受所干扰？(PG) | 0.10 | **0.82** | -0.02 | -0.04 |
| 6.为了持续感受消极情绪（如生气，悲伤）？(CHG) | 0.05 | -0.18 | **0.85** | -0.04 |
| 5.为了感受更少的积极情绪（如高兴，满足）？(CHG) | -0.03 | 0.05 | **0.77** | 0.05 |
| 4.为了感受更多的消极情绪（如生气，悲伤）？(CHG) | 0.09 | -0.11 | **0.77** | -0.01 |
| 3.为了持续感受积极情绪（如高兴，满足）？(PHG) | 0.20 | 0.12 | 0.05 | **0.87** |
| 2.为了感受更多的积极情绪（如高兴，满足）？(PHG) | 0.13 | 0.20 | -0.04 | **0.86** |

NOTE: PHG = pro-hedonic goals; CHG = contra-hedonic goals; PG = performance goals; PSG = pro-social goals; IMG = impression management goals. Boldface indicates items’ scale membership as determined by factor loading.

**S-Table 3** **Five-Factor Loading Matrix after item 1 removal (NA =917)**

| *ERGS Item* | Factor | | | | |
| --- | --- | --- | --- | --- | --- |
|  | 1 | 2 | 3 | 4 | 5 |
| 18.为了给他人留下一个好的印象？（IMG) | **0.74** | 0.21 | -0.03 | 0.34 | -0.12 |
| 17.为了得到他人的认可？(IMG) | **0.72** | 0.28 | -0.07 | 0.37 | -0.07 |
| 12.为了避免与他人疏远？(PSG) | **0.72** | 0.26 | -0.02 | -0.26 | 0.003 |
| \| 16.为了避免给他人留下一个令人不快的印象？(IMG) \| \| --- \| | **0.72** | 0.16 | -0.01 | 0.25 | -0.15 |
| \| 15.为了避免被他人拒绝？(IMG) \| \| --- \| | **0.67** | 0.34 | -0.03 | 0.16 | -0.07 |
| \| 14.为了与他人保持亲密关系？(PSG) \| \| --- \| | **0.66** | 0.24 | -0.06 | 0.04 | 0.04 |
| 10.为了使他人感到舒适？(PSG) | **0.66** | 0.15 | -0.01 | -0.41 | 0.10 |
| 13.为了使他人开心？(PSG) | **0.65** | 0.32 | -0.05 | -0.22 | 0.06 |
| 11.为了避免破坏他人的心情？(PSG) | **0.64** | 0.21 | 0.03 | -0.43 | 0.03 |
| 4.为了感受更多的消极情绪（如生气，悲伤）？(CHG) | -0.23 | **0.66** | **0.50** | 0.10 | 0.23 |
| 8.为了能够专注于自己的工作或正在进行的事情？(PG) | 0.41 | **-0.62** | **0.63** | -0.03 | -0.13 |
| 6.为了持续感受消极情绪（如生气，悲伤）？(CHG) | -0.28 | **0.60** | 0.49 | 0.05 | 0.26 |
| 2.为了感受更多的积极情绪（如高兴，满足）？(PHG) | 0.41 | **-0.56** | -0.10 | 0.11 | 0.39 |
| 9.为了能够保持对所进行工作的专注？(PG) | 0.41 | **-0.56** | **0.57** | -0.04 | -0.09 |
| 5.为了感受更少的积极情绪（如高兴，满足）？(CHG) | -0.21 | **0.52** | 0.39 | 0.06 | 0.06 |
| 7.为了避免被自己的情绪感受所干扰？(PG) | 0.27 | -0.44 | 0.34 | 0.02 | -0.12 |
| 3.为了持续感受积极情绪（如高兴，满足）？(PHG) | 0.45 | -0.46 | -0.10 | 0.18 | **0.55** |

NOTE: PHG = pro-hedonic goals; CHG = contra-hedonic goals; PG = performance goals; PSG = pro-social goals; IMG = impression management goals. Boldface indicates items’ scale membership as determined by factor loading.
